# Screening Equine Skin and Mucous Membranes for Carriage of Coagulase‐Positive Staphylococci

**DOI:** 10.1111/vde.70099

**Published:** 2026-06-25

**Authors:** Christina M. Dierkes, Daniel O. Morris, Christine L. Cain, Laurel E. Redding, Maho Okumura, Alison Gore, Stephen D. Cole

**Affiliations:** ^1^ Department of Clinical Sciences & Advanced Medicine School of Veterinary Medicine, University of Pennsylvania Philadelphia Pennsylvania USA; ^2^ Department of Clinical Studies – New Bolton Center School of Veterinary Medicine, University of Pennsylvania Philadelphia Pennsylvania USA; ^3^ Department of Pathobiology School of Veterinary Medicine, University of Pennsylvania Philadelphia Pennsylvania USA

## Abstract

**Background:**

Commensal populations of coagulase‐positive *Staphylococcus* spp. (CoPS) in healthy horses are not well‐defined.

**Objective:**

To determine the point prevalence, anatomical distribution, antimicrobial susceptibility and genomic characteristics of CoPS in healthy horses.

**Animals:**

One‐hundred and fifty privately owned, systemically healthy horses from seven facilities were enrolled in this study.

**Materials and Methods:**

Horses were sampled from the nares, buccal mucosa and three skin sites. Isolates were identified using matrix‐assisted laser desorption/ionisation time‐of‐flight mass spectrometry, and antimicrobial susceptibility testing was performed by gradient diffusion. Whole‐genome sequencing with multilocus sequence typing (MLST) characterised *Staphylococcus aureus* lineages. *In silico* PCR differentiated Group A and Group B *Staphylococcus delphini* . Sampling site sensitivity was calculated using a multisite composite reference. Mixed‐effects logistic regression evaluated associations between demographic factors and carriage.

**Results:**

Carriage of any staphylococcal species occurred in 111 of 150 (74%) horses: *S. aureus* 28 of 150 horses (18.7%) and *S. delphini* 23 of 150 horses (15.3%). Increasing age was associated with decreased odds of CoPS isolation (odds ratio 0.92/year; 95% confidence interval 0.86–0.98). The nares were the most sensitive single sampling site, whereas multisite sampling improved detection. Meticillin–resistance was absent in all CoPS isolates, and *S. delphini* isolates were fully susceptible to all antimicrobials tested. MLST revealed multiple *S. aureus* sequence types, some of which were shared across multiple horses at some boarding facilities. Both groups of *S. delphini* lineage were identified, although many isolates were nontypable.

**Conclusions and Clinical Relevance:**

Healthy horses harbour *S. aureus* and *S. delphini* , with minimal antimicrobial resistance. Shared *S. aureus* sequence types within facilities suggest possible lateral transmission.

## Abstract 2

**Background:** Commensal populations of coagulase‐positive *Staphylococcus* spp. (CoPS) in healthy horses are not well defined. **Objective:** To determine the point prevalence, anatomical distribution, antimicrobial susceptibility and genomic characteristics of CoPS in healthy horses. **Conclusions and Clinical Relevance:** Healthy horses harbour *S. aureus* and *S. delphini* , with minimal antimicrobial resistance. Shared *S. aureus* sequence types within facilities suggest possible lateral transmission.

## 1 Introduction

Staphylococci are commensal inhabitants of the surfaces of healthy skin and mucous membranes, and also serve as the primary agents of skin and soft tissue infections of domestic animals [1]. It has been clearly demonstrated through molecular strain typing techniques that canine pyoderma is usually caused by the same strain(s) of *Staphylococcus pseudintermedius* that normally colonise the dog's carriage sites such as the mouth, nose or skin [2, 3, 4]. This also is the case for *S. aureus* infections of people [5], yet a similar relationship has not yet been described for horses. However, equine pyoderma appears to be associated with a more diverse array of *Staphylococcus* spp. than in dogs or people [6].

Prior studies of equine colonisation by staphylococci have purposefully selected for meticillin‐resistant *Staphylococcus* (MRS) strains, and most have focused exclusively on selection for MR *S. aureus* (MRSA) [7]. The primary goals of most studies published to date have been to define the role of horses as potential reservoirs of MRSA and/or to define risk factors for MRS acquisition in hospitalised horses. As these studies have selectively reported prevalence of MRS at carriage sites rather than carriage of any or all staphylococci of pathogenic potential, the prevalence of colonisation by all species of coagulase‐positive *Staphylococcus* spp. (CoPS) in healthy horses has not been characterised. In healthy donkeys, *S. delphini* dominates, having been isolated from the nares of 19% of 100 donkeys by nasal swab [8].

Therefore, it has been unclear which of the CoPS are truly commensal organisms in horses. Additionally, rates of antimicrobial resistance to drugs other than beta lactams may have been overestimated by these prior studies because they focused exclusively on testing MRS isolates. It has been widely documented that most MRS strains will be concurrently multidrug resistant (MDR) [1, 7]. Therefore, there is a need to estimate antimicrobial resistance rates in staphylococci isolated from a general population of horses where all isolates are characterised. This information would help to inform empirical prescribing practices.

A prerequisite to defining staphylococcal carriage in any animal species is an understanding of which potential carriage sites must be sampled. In dogs, the oral cavity has been shown by at least three independent studies [4, 9, 10] to be the single most sensitive site for defining carriage, yet sensitivity is improved by including the perineum. In horses, a single nare has been the site screened for MRS by most studies published to date. However, one MRSA screening study showed that sampling both nares improved sensitivity of detection [11] and another showed that including a least one skin site (from either the carpus, neck, withers or croup) also improved sensitivity [12]. Based on these reports, screening studies which aim to define equine colonisation should include both nares and at least one skin site in the sampling strategy. The value of the oral mucosa (buccal space) remains to be defined in horses.

The objectives of this study were to: (a) define the relative prevalence rates of CoPS carriage (skin and mucous membranes), (b) define the most sensitive carriage site (or combination of carriage sites) for future screening studies of horses; (c) define the antimicrobial susceptibility patterns of CoPS isolated from systemically healthy horses; and (d) conduct whole‐genome sequencing (WGS) to define clonal relationships of the isolates. We hypothesised that (i). *S. delphini* would be the most commonly isolated species, with other CoPS (*S. aureus, S. pseudintermedius* and *S. hyicus* ) being isolated at lower rates and in association with environmental exposures; (ii) sampling of both nares plus skin sites would provide the highest sensitivity, with buccal mucosa having less value than in dogs; (iii) antimicrobial resistance would be uncommon in CoPS isolates obtained from healthy horses without recent exposure to veterinary care or antimicrobial use; and (iv) WGS would identify expansion of *S. aureus* clones across horses residing in a broad geographical area, and which had no contact with each other.

## 2 Materials and Methods

### 2.1 Ethical Oversight and Consent

This study protocol was approved by the University of Pennsylvania Institutional Animal Care and Use Committee (IACUC). Equine owners (or authorised agent) gave written consent via signature on a consent form before sample collection.

### 2.2 Owner Survey

Owners of horses (or their designated/authorised agents) completed a comprehensive survey regarding each horse's demographic data (age and sex); housing (stabled, partial turnout and pastured); length of time at current facility and travel to other facilities; contact with other horses and other animal species; contact with veterinary facilities/veterinary personnel; contact with people who work in human healthcare or who have been hospitalised recently; exposures to systemic and topical antimicrobial agents; and history of any skin disease. The questionnaire can be viewed in Supporting Information Appendix S1.

### 2.3 Sample Size

As no prior data were available regarding prevalence of CoPS carriage in healthy horses, sample size was based on the goal of obtaining ≥ 30 isolates of each relevant CoPS species. As per Clinical and Laboratory Standards Institute (CLSI) guidelines, ≥ 30 isolates is recommended when constructing composite antibiograms [13]. Therefore, the total number of horses to be sampled was extrapolated from the isolation rates documented in the first 50 subjects. Using these preliminary results, we predicted that a total of 150 horses would be required to achieve the ‘30‐isolate’ goal.

### 2.4 Sample Population

In order to capture a comprehensive range of factors influencing the transfer and carriage of *Staphylococcus* bacteria in horses, this study included horses from diverse housing environments, each representing different potential exposures to people and other horses. The majority of horses were selected for distinct backgrounds, each with unique characteristics that could affect bacterial dynamics:

1. **Large boarding and riding lesson facility**: This facility housed > 40 of the horses, each individually stalled and turned out in either private or group paddocks, with some horses sharing a fence line. The facility experiences frequent human traffic, with barn staff and members of the public regularly interacting with the horses.
2. **Rescue facility**: The rescue houses horses of varied backgrounds and provides full turnout in group paddocks, some of which share fence lines. Volunteers and barn employees interact with these horses, contributing to potential bacterial exposure and transfer.
3. **Therapeutic riding centre**: Horses at this facility are individually stalled and turned out in private paddocks, with some shared fence lines. Similar to the other locations, there is regular interaction with barn employees and members of the public.
4. **Private facilities**: Horses housed in private facilities, where human interaction and exposure to environmental variables were minimal compared with the group housing locations, also were included.

In order to control for confounding factors, horses that were actively receiving antimicrobial treatment or suffering from any type of skin lesions at the time of sampling were excluded from the study. These exclusions ensured that bacterial carriage data accurately reflected natural patterns of staphylococcal presence, without interference from medical treatments or active skin conditions. By selecting horses from diverse settings, we sought perspective on how housing conditions and human interactions might influence *Staphylococcus* carriage in healthy horses. However, the study was not powered to assess staphylococcal transmission dynamics, so evaluation of these factors was for exploratory purposes only.

### 2.5 Sampling Procedures

Flocked polyester‐tipped swabs were taken from the mouth, both nares and skin surface (neck, one axilla, groin area) of 150 systemically healthy, privately owned equines on their own premises in southeast Pennsylvania between August 2024 and May 2025. The nare swab was passed into the nare to the depth of 10 cm, rotated, and withdrawn. For the first 50 horses, both nares were sampled separately. When it was noted that no horse had different CoPS species isolated from each nare, both nares of the remaining horses were sampled with the same swab to reduce the cost of sample processing. The buccal mucosa swab was introduced at the corner of the mouth, rubbed between the horse's cheek and gums for 5 s, and withdrawn. One swab was used to rub the skin (for 10 s) at each of the following sites and in the following order: neck (under the mane; a human ‘touch’ zone), one axilla and the groin area. The same swab was used for all three sites.

### 2.6 Isolation and Identification of Staphylococci

Each swab was processed by the microbiology laboratory using brain–heart infusion broth enrichment to first enhance growth and then selected for isolation of *Staphylococcus* spp. on colistin‐nalidixic acid (CNA) agar. Subculture of up to three colonies for each unique morphology on the initial culture was performed and all unique morphologies were speciated (e.g., if four morphologies were present then 12 isolates were characterised). Speciation was achieved with matrix‐assisted laser desorption/ionisation time‐of‐flight (MALDI‐TOF; Sirius, Bruker). Oxacillin and cefoxitin susceptibility were then assessed by Kirby–Bauer disc diffusion to define meticillin–resistance (MR) status according to CLSI standards [14]. All isolates (including both CoPS *and* coagulase‐negative species) were banked at −70°C in a commercial storage system (Microbank cryopreservation tubes; Prolab Diagnostics) for future processing.

### 2.7 Antimicrobial Susceptibility Testing

Cefoxitin disc diffusion was performed to screen for MR on all CoPS isolates.

Isolates were determined to be resistant after appropriate incubation for the determined species (16–18 h for *S. aureus* or 24 h for *S. delphini* ) according to CLSI standards [14]. PBP2a lateral flow assay was performed to ultimately determine the MR status. Additional antimicrobial susceptibility testing (AST) was performed for one CoPS isolate of each species (e.g., *S. delphini* and *S. aureus* ), per horse. When more than one anatomical site was positive for the same CoPS species, the nasal isolate was prioritised for AST and WGS. Buccal isolates were used for AST and WGS when the nasal swab was negative. Skin isolates were used when they represented the only positive site for the CoPS species in question. AST was performed by gradient agar diffusion (Liofilchem) on Mueller–Hinton agar (Remel) according to the manufacturers' instructions. This method was selected because current commercial broth microdilution panels are not inclusive of appropriate dilutions to test several current CLSI breakpoints established for staphylococci isolated from horses. Quality control was performed for each batch of AST performed using *S. aureus* (ATCC 25923). AST was performed for drugs with established CLSI equine breakpoints (amikacin, enrofloxacin and minocycline) as well as trimethoprim‐sulfamethoxazole (TMS) given its prevalent use and widely applied human breakpoint.

### 2.8 Sequencing

Sequencing was performed on *S. delphini* and *S. aureus* isolates using a commercial sequencing laboratory (SeqCenter, Pittsburgh, PA, USA). Briefly, DNA was extracted from pure bacterial colonies suspended in sterile lysis solution according to the kit's manufacturer's instructions (ZymoBIOMICS DNA Miniprep Kit). Libraries were prepared using the tagmentation‐based and PCR‐based Illumina DNA Prep Kit and custom IDT 10‐bp unique dual indices (UDI) with a target insert size of 280 bp. No additional DNA fragmentation or size selection steps were performed. Illumina sequencing was performed on an NovaSeq X Plus sequencer in one or more multiplexed shared‐flow‐cell runs, producing 2 × 151‐bp paired‐end reads. Demultiplexing, quality control and adapter trimming was performed with bcl‐convert1 (v4.2.4). Sequencing statistics are included in the ‘DNA Sequencing Stats.xlsx’ file. Raw reads that met quality control standards were assembled to draft genomes using the Pathogen Genome Annotation Pipeline (BV‐BRC) using default parameters. Multilocus sequence typing was performed using default parameters for *S. aureus* using MLST [15] and resfinder (v4.7.2) tools [16]. *In silico* PCR was performed using default parameters and previously published primers to differentiate between Group A and Group B *S. delphini* [17, 18].

### 2.9 Statistical Analysis

The prevalence of each species and combination of species of *Staphylococcus* was calculated.

Carriage was defined as a horse having at least one culture‐positive sampling site for any *Staphylococcus* species. To assess the association of horse‐ and barn‐level factors with the likelihood of colonisation with any *Staphylococcus* species, univariable mixed‐effects logistic regression models were fitted at the horse level, with facility included as a random effect and each of the variables age, sex, turnout, time at facility, recent travel off‐premises, oral supplements, recent veterinary treatment, exposure to a veterinary hospital, recent antibiotic therapy, recent skin infections and any exposure of the owner to a healthcare system as fixed effects. This analysis was exploratory and intended to be hypothesis‐generating (rather than hypothesis‐testing) given the relatively low prevalence of isolates and some demographic factors.

Collinearity among candidate variables was assessed; then, variables that were associated (*p* < 0.15) with the outcome on univariable analysis were added to a multivariable model in a stepwise fashion and retained if they remained significantly associated (*p* < 0.05) with the outcome. The linearity of ‘age’ in the logit model was assessed using increasingly flexible specifications (quadratic and splines). Neither the spline model nor the quadratic model significantly improved fit over the linear specification (LR test/AIC/BIC). Therefore, age was modelled linearly for parsimony.

In order to evaluate the relative sensitivity of individual sampling sites for detecting *Staphylococcus* colonisation, we compared detection rates at each site to a reference standard (a composite of all sites). Because no true gold standard exists for detecting *Staphylococcus* colonisation in horses, carriage was confirmed if any sampled site yielded a positive culture. Under this definition, combined sampling of the nares, oral cavity and skin was assumed to represent 100% detection. The relative sensitivity of each individual sampling site was then calculated as the proportion of colonised horses (i.e., positive at any site) that were positive at that specific site. This approach estimates the relative contribution of each sampling site to overall detection rather than true diagnostic sensitivity.

## 3 Results

A total of 150 horses residing at seven locations were analysed for *Staphylococcus* carriage. Of these, 39 (26%) were negative for any *Staphylococcus* species, whereas 111 (74%) were positive for at least one species of *Staphylococcus*. The distribution of different *Staphylococcus* species isolated from individual horses is summarised in Table 1. The ‘other Staph’ category included two horses with *S. hyicus* , and 65 horses with *Mammaliicoccus sciuri* (formerly *S. sciuri* ) [19]. The remainder of this category were various coagulase‐negative *Staphylococcus* spp. (CoNS). Coagulase‐negative staphylococci were not analysed further, nor were their distributions through the sample population characterised epidemiologically.

**TABLE 1:** Distribution of *Staphylococcus* spp. across 150 horses.

*Staphylococcus* species	*N* (%)
No *Staphylococcus* spp.	39 (26%)
Any *Staphylococcus* spp.	111 (74%)
*S. aureus*	28 (18.7%)
*S. delphini*	23 (15.3%)
*S. aureus* only	7 (4.7%)
*S. delphini* only	6 (4.0%)
*S. aureus* + *S. delphini*	2 (1.3%)
*S. aureus* + other Staph	15 (10%)
*S. delphini* + other Staph	8 (5.3%)
*S. aureus* + *S. delphini* + other Staph	3 (2%)
Other Staph only^a^	66 (44%)

^a^ Includes two horses from which *S. hyicus* was isolated, and all *Mammaliicoccus sciuri* isolates.

The prevalence of isolation of each *Staphylococcus* species varied across sampling sites (nares, mouth and skin sites). The nares were the most commonly positive site for all *Staphylococcus* species, particularly for CoNS (29% of horses) and *S. aureus* (42.9% of horses). The sampling site breakdown for each *Staphylococcus* species is presented in Table 2.

**TABLE 2:** Sampling site breakdown for each *Staphylococcus* species.

	Skin	Mouth	Nares	Nares + mouth	Mouth + skin	Nares + skin	All sites
*S. aureus*	7 (25%)	7 (25%)	12 (42.9%)	2 (7.1%)			
*S. delphini*	3 (13%)	1 (4.3%)	12 (52.2%)	3 (13%)	1 (4.3%)	3 (13%)	
Other Staph	5 (5.4%)	6 (6.5%)	27 (29%)	14 (15.1%)	3 (3.2%)	11 (11.8%)	27 (29%)

A mixed‐effects logistic regression, considering location as a random effect, identified age as the only statistically significant factor associated with *Staphylococcus* colonisation. Specifically, for every 1‐year increase in age, the odds of colonisation with any *Staphylococcus* species decreased by 8% (odds ratio [OR] = 0.92, *p* = 0.021, 95% confidence interval [CI] = 0.86–0.98). This association was consistent for both *S. aureus* (OR = 0.93, *p* = 0.038, 95% CI = 0.87–0.99) and *S. delphini* (OR = 0.93, *p* = 0.036, 95% CI = 0.87–0.99). No significant associations were observed for ‘Other Staph’ species. When analysing using standard logistic regression (without random effects), partial turnout (stall + turnout) was associated with an increased risk of colonisation with any *Staphylococcus* species (OR = 2.4, *p* = 0.028, 95% CI = 1.1–5.2) relative to 24/7 turnout. However, this result is likely to have been confounded by location, as only one location employed 24/7 turnout, whereas all other locations employed partial turnout.

The sensitivity of various sampling sites for detecting *Staphylococcus* species was evaluated, using ‘positive by any site’ as the gold standard. The results are summarised in Table 3. Overall, the nares were the most sensitive site for detecting *Staphylococcus* species, particularly for ‘Other Staph’ (84%), followed by *S. delphini* (78%) and *S. aureus* (50%). Oral and skin sampling had lower sensitivity for all species, with oral sampling showing the highest sensitivity for ‘Other Staph’ (60%) and skin sampling being the least sensitive for *S. aureus* . The sensitivity of ‘nose+mouth+skin’ will always be 100% and therefore always the ‘most sensitive option’. These data are presented in Table 3.

**TABLE 3:** Sensitivity site detection.

*Staphylococcus* type	Nares sensitivity (%)	Mouth sensitivity (%)	Skin sensitivity (%)
*S. aureus*	50% (14/28)	32% (9/28)	25% (7/28)
*S. delphini*	78% (18/23)	22% (5/23)	30% (7/23)
Other Staph	84% (79/66)	60% (56/66)	49% (46/66)

### 3.1 Antimicrobial Susceptibility Testing

None of the *S. delphini* or *S. hyicus* isolates exhibited resistance to any of the antimicrobial drugs tested. Of the *S. aureus* isolates, one was resistant to TMS, 11 were of intermediate susceptibility to minocycline, three were of intermediate susceptibility to both minocycline and enrofloxacin, and one was of intermediate susceptibility to only enrofloxacin. Cefoxitin screening was positive for three *S. delphini* isolates; however, these isolates were subsequently negative by PBP2a testing and for the *mecA* gene by WGS (see below). All isolates were therefore determined to be meticillin‐susceptible (MS).

### 3.2 Whole‐Genome Sequencing

Species identification was confirmed by WGS and k‐mer analysis. All isolates also were confirmed to not include *mecA*. To assess the genetic diversity of representative isolates from the collection, we performed short read WGS and subsequent *in silico* typing. The 28 isolates of *S. aureus* were characterised as ST1181 (*N* = 10), ST133 (*N* = 7), ST1660 (*N* = 3), ST1 (*N* = 3), ST6615 (*N* = 2) and ST45 (*N* = 1). Two additional isolates were characterised as unknown yet had identical allelic profiles (*arcC*‐1, *aroE*‐1, *glpF*‐1, *gmk*‐1, *pta*‐1, *tp*‐1 and *yqiL*523). Relationships between horses from which *S. aureus* clones were isolated are summarised in Table 4. Briefly, three *S. aureus* strains (ST1181, ST133 and ST1660) were identified from horses across multiple sites, and all strains other than ST45 were isolated from two or more horses within at least one facility. For *S. delphini* , four isolates were Group A, nine were Group B and the remainder were nontypeable using the established scheme.

**TABLE 4:** Distribution of *Staphylococcus aureus* clones by geographical location.

Strain type	Location 1	Location 2	Location 6	Location 7
ST 1181 (*N* = 10)	N/A	Six horses	Three horses	One horse
ST 133 (*N* = 7)	One horse	Two horses	Four horses	N/A
ST 1660 (*N* = 3)	N/A	N/A	One horse	Two horses
ST 1 (*N* = 3)	N/A	N/A	N/A	Three horses
ST 6615 (*N* = 2)	N/A	N/A	Two horses	N/A
ST 45 (*N* = 1)	N/A	One horse	N/A	N/A

## 4 Discussion

This study aimed to characterise the prevalence of CoPS carriage on the skin and mucous membranes of healthy horses and to construct composite antibiograms for each CoPS species isolated. Our results revealed that *S. aureus* and *S. delphini* were the most prevalent CoPS species, with 29 of 150 (18.7%) and 23 of 150 (15.3%) of horses being carriers, respectively. Notably, neither *S. pseudintermedius* nor *S. coagulans* —the dominant CoPS species involved in canine colonisation and infection—were isolated. A single isolate of *S. hyicus* , which is the predominant CoPS species carried by swine [20], was recovered from the mouth of one horse.

We did not achieve our goal of testing 30 isolates of each CoPS species (sample size based on the rate of isolation within the first 50 horses), yet composite antibiograms showed that *S. delphini* was fully susceptible to all antimicrobials tested. None of the CoPS isolates were MR as confirmed by WGS (absence of a *mec*A gene). Notably, three *S. delphini* isolates were positive by cefoxitin screen before a negative PBP2a test and WGS confirmed these isolates to be MS. This suggests a potentially high false‐positivity rate for this bacterial species using the current CLSI recommended methodology although further studies are needed. Cefoxitin is known to perform poorly for some bacterial species and this may be true for *S. delphini* as well [21]. A total of 14 of the *S. aureus* isolates were of intermediate susceptibility to minocycline, and one isolate was resistant to TMS. Both of these antimicrobials are commonly used for the empirical treatment of staphylococcal infections of horses; therefore, AST should be recommended whenever feasible to guide drug selection.

This study identified age as a factor significantly associated with CoPS, with older horses showing a decreased likelihood of carrying either *S. aureus* or *S. delphini* . The inverse relationship between age and staphylococcal carriage in horses may reflect the development of immunity over time or changes in environmental exposure, although this hypothesis requires further investigation.

No other demographic factors, such as type of housing, grouping by facility or boarding type, contact with other animals or exposure to antimicrobials or veterinary care were found to be associated with carriage. Failure to detect such associations may be reflective of an underpowered study, because the sample size was not powered with these goals in mind. Additionally, previous studies have found antimicrobial exposure to be a key driver for MRSA carriage in hospitalised horses [11, 12, 22, 23], whereas our study focused exclusively on systemically healthy horses from private farms and boarding facilities.

Genetic analysis of selected isolates revealed clustering of *S. aureus* clones among some horses residing at the same premises. These findings suggest that localised transmission of *S. aureus* may occur despite the absence of statistically significant associations identified through conventional epidemiologic modelling. Again, though, the study was likely to have been underpowered for detecting such associations. Transmission could be facilitated through indirect contact mechanisms, including shared equipment (e.g., grooming tools, tack and feed buckets) or human‐mediated transfer via handlers, veterinary personnel or barn staff. Most of the horses sampled were housed in large boarding facilities with multiple riders receiving lessons, rescues with exposure to multiple volunteers or therapeutic barn centres with lessons and volunteers. Although the study was likely to have been underpowered for detecting subtle management‐associated effects, strain‐level genetic relatedness suggests that microenvironmental or management‐specific factors could still influence bacterial dissemination within individual facilities. Further studies incorporating detailed contact‐network analysis would be valuable to better characterise transmission dynamics of CoPS in equine populations. Future studies should be powered to integrate genotypic data with traditional risk factor analyses when investigating equine staphylococcal epidemiology.

Clonal inferences for *S. delphini* isolates were limited to *in silico* PCR analysis of WGS data. The typing scheme described for *S. delphini* segregates isolates into two groups: A and B. Of the 23 isolates examined, 10 could not be typed by this scheme, highlighting the need for the development of more robust genetic typing schemes for this species.

We also sought to determine the most sensitive carriage sites for screening. In horses, single‐site sampling, particularly from one nare, has often been used for MRSA screening [21]. In our healthy horse population, sampling of both nares independently did not yield disparate CoPS results for any of the first 50 horses sampled, although six of these were positive from only one nare. Therefore, sampling both nares with a single swab is recommended to contain costs of processing. The skin sites were chosen to represent two intertriginous areas (axilla and groin) where the hair is sparse, and one ‘high touch’ site (lateral neck) where human contact may be greater. In dogs, intertriginous areas are common sites for staphylococcal colonisation and pyoderma lesions [10]. Additionally, previous studies in dogs have demonstrated that the oral cavity is the most sensitive site for staphylococcal detection [9, 10]. We found that sampling the oral cavity and skin sites of horses added value, even though sensitivities were low compared with the nares. Nine horses were positive for CoPS only on buccal swabs (including one with *S. hyicus* ), whereas 10 horses were positive for CoPS only on skin swabs. Therefore, to maximise the detection of CoPS carriage in horses, sampling both nares, the oral cavity and a composite of skin sites (neck, axilla and groin) is recommended.

A major limitation of this study was its cross‐sectional design, which estimated carriage of CoPS at only one point in time. Future studies should employ a longitudinal sampling design with multiple time points, in order to better estimate true *colonisation* by self‐perpetuating populations of CoPS.

The point prevalence rates of CoPS carriage in our study support the conclusions that *S. aureus* and *S. delphini* are the primary staphylococcal species in healthy horses. These findings are consistent with some reports of staphylococcal diversity in horses, where *S. aureus* and *S. delphini* were commonly isolated from skin lesions of horses with pyoderma [11, 12, 23]. Our findings suggest that antimicrobial resistance within CoPS of healthy horses in community settings, at least in the geographical region studied, is uncommon yet could conceivably impact drug selection when considering empirical treatment of a suspected staphylococcal infection.

## Author Contributions

**Christina M. Dierkes:** writing – original draft, resources, conceptualization. **Christine L. Cain:** conceptualization, writing – review and editing. **Laurel E. Redding:** data curation, software, formal analysis. **Alison Gore:** data curation. **Stephen D. Cole:** conceptualization, writing – review and editing, data curation, supervision, software, formal analysis. **Daniel O. Morris:** conceptualization, writing – review and editing, data curation, resources, methodology. **Maho Okumura:** data curation.

## Funding

This work was supported by the American College of Veterinary Dermatology and the Animal Dermatology Group.

## Conflicts of Interest

Daniel Morris is EiC, Veterinary Dermatology and was excluded from the peer‐review process for this manuscript.

## Supporting information


**Appendix S1:** Supporting Information.

## Data Availability

The data that support the findings of this study are available from the corresponding author upon reasonable request.
