# Supplementary material for: Screening Equine Skin and Mucous Membranes for Carriage of Coagulase‐Positive Staphylococci
Source: Vet Dermatol. 2026 Jun 25;37(5):681–7. doi: 10.1111/vde.70099 (PMC13569202; doi:10.1111/vde.70099)
Supplement: Supplementary file 1 [file VDE-37-681-s001.docx]

**Please complete this form and return it to *(removed for anonymity)**

1. How is this horse housed? □ Full turnout; □ Partial turnout; □ Fully stabled
   a. If turned out, is there standing water near or around the paddock? Y/N
   b. If stabled, how often is the stall cleaned? __________
2. How long has this horse been boarding at its current facility? □ <1 year; □ 1–3 years; □ >3 years
3. Does this horse have direct contact with other horses in the barn/facility? Y/N
4. Has this horse traveled to other facilities within the last 4 months? For example: horse shows, training, veterinary hospital, breeding facility, etc. Y/N
5. Is this horse on oral supplements? Y/N
   a. If yes, what supplements? __________
6. Has this horse been treated by a veterinarian at your facility within the past 4 months? Y/N
7. Has this horse been hospitalized at a veterinary treatment facility within the past 4 months? Y/N
8. Has this horse been treated with antibiotic medications within the last 4 months? Y/N
   a. If yes, do you know which drug? __________
9. Does this horse have a history of skin disease? Y/N
   a. If yes, what has this horse been diagnosed with? __________
   b. If yes, does this horse also have a history of skin infections? Y/N
   c. If yes, do you know which type of infection (examples: Staph. bacteria, ringworm fungus, “scratches”, etc.?) __________
10. Have any antiseptic (chlorhexidine, iodine, hydrogen peroxide, etc.) based topical grooming and/or fly control products been used on this horse within the last month? And, at what frequency?
    a. Shampoos/conditioners: _________________________, ____ times per week
    b. Fly sprays: _________________________, ____ times per week
    c. Coat/Wound care: _________________________, ____ times per week
    d. Other: _________________________, ____ times per week
11. Is this horse owned by or does it come into contact with a human healthcare provider (for example: a doctor, nurse, physical therapist, dental hygienist, etc.)? Y/N/Unknown
12. Has any person in contact with this horse been hospitalized within the last 4 months? Y/N/Unknown
